# Mosaicism of Mitochondrial Genetic Variation in Atherosclerotic Lesions of the Human Aorta

**DOI:** 10.1155/2015/825468

**Published:** 2015-03-05

**Authors:** Margarita A. Sazonova, Vasily V. Sinyov, Valeria A. Barinova, Anastasia I. Ryzhkova, Andrey V. Zhelankin, Anton Y. Postnov, Igor A. Sobenin, Yuri V. Bobryshev, Alexander N. Orekhov

**Affiliations:** ^1^Laboratory of Angiopathology, Institute of General Pathology and Pathophysiology, 8 Baltiyskaya Street, Moscow 125315, Russia; ^2^Laboratory of Medical Genetics, Russian Cardiology Research and Production Complex, 15a 3rd Cherepkovskaya Street, Moscow 121552, Russia; ^3^Faculty of Medicine, School of Medical Sciences, University of New South Wales, Sydney, Kensington, NSW 2052, Australia; ^4^School of Medicine, University of Western Sydney, Campbelltown, NSW 2560, Australia; ^5^Institute for Atherosclerosis Research, Skolkovo Innovation Centre, Moscow 121552, Russia

## Abstract

*Objective.* The aim of the present study was an analysis of heteroplasmy level in mitochondrial mutations 652delG, A1555G, C3256T, T3336C, 652insG, C5178A, G12315A, G13513A, G14459A, G14846A, and G15059A in normal and affected by atherosclerosis segments of morphologically mapped aortic walls. *Methods.* We investigated the 265 normal and atherosclerotic tissue sections of 5 human aortas. Intima of every aorta was divided according to morphological characteristics into segments with different types of atherosclerotic lesions: fibrous plaque, lipofibrous plaque, primary atherosclerotic lesion (fatty streak and fatty infiltration), and normal intima from human aorta. PCR-fragments were analyzed by a new original method developed in our laboratory on the basis of pyrosequence technology. *Results.* According to the obtained data, mutations G12315A and G14459A are significantly associated with total and primary atherosclerotic lesions of intimal segments and lipofibrous plaques (*P* ≤ 0.01 and *P* ≤ 0.05, accordingly). Mutation C5178A is significantly associated with fibrous plaques and total atherosclerotic lesions (*P* ≤ 0.01). A1555G mutation shows an antiatherosclerotic effect in primary lesion in lipofibrous plaques (*P* ≤ 0.05). Meanwhile, G14846A mutation is antiatherogenic for lipofibrous plaques (*P* ≤ 0.05). *Conclusion.* Therefore, mutations C5178A, G14459A, G12315A, A1555G, and G14846A were found to be associated with atherosclerotic lesions.

## 1. Introduction

Atherosclerosis of great vessels in the overwhelming majority of cases is predominantly a morphological basis of cardiovascular mortality [1, 2]. In the twenty-first century, atherosclerosis began to be of epidemical nature [2]. Previously, this pathology was specific only for elderly people, but now it affects even young individuals. The significance and timeliness of investigation of the pathology seem obvious.

A specific feature of atherosclerosis is a complicacy of this disease detection at early stages. Molecular-genetic markers associated with atherosclerosis can help early diagnosis of this pathology. In recent times, a great number of studies were dedicated to searching genetic biomarkers of atherosclerosis and CVD in human nuclear genome [3–5]. However, nuclear genome mutations have a rather low diagnostic and prognostic significance compared to certain traditional risk factors of atherosclerosis and cardiovascular diseases. A relative risk of each known polymorphism associated with atherosclerosis or CVD is 1.06–1.40. A total risk of cardiovascular disease presence for known nuclear genome polymorphisms is approximately 5% [6].

According to literature data, various pathologies are associated with certain mitochondrial mutations [7–9]. Mitochondrial genome mutations correlate with different diseases such as diabetes, some forms of deafness, myopathy, coronary vessel stenosis, predisposition to acute myocardial infarction, and cardiomyopathy. These pathologies often occur together with atherosclerosis [7–9].

Investigations of researchers all over the world are mainly dedicated to autosomal mutations, associated with atherosclerosis [10–12]. Only several works are dedicated to molecular-genetic defects of mitochondrial genome associated with atherosclerotic lesions [13–15]. In most of such investigations, large-scale deletions, leading to a full dysfunction of mitochondrial genome, were analyzed [16–18].

It is necessary to mention that because of mitochondrial genome instability, somatic mutations often occur in mitochondrial genome. While analyzing the association of mitochondrial genome with pathologies, it is necessary to carry out a quantitative assessment of mitochondrial genome heteroplasmy level [19–21].

In the present study, an association of eleven mitochondrial genome mutations with atherosclerosis was analyzed in individuals, aortic intima of which was morphologically divided into segments with atherosclerotic lesions of varying severity.

## 2. Materials and Methods

### 2.1. Materials

265 normal and atherosclerotic segments of morphologically mapped aortic walls of varying severity of five individuals were taken as a material for investigation.

Samples of autopsy material were taken from thoracic section of aortic intima of men and women, who died at the age of 30–65 years as a result of an accident or a sudden death (except acute alcoholic and other intoxications and electrical injury).

To determine a type of a lesion, normal segments of aortas and segments with atherosclerotic lesions were identified macroscopically and then microscopically in accordance with classification of Atherosclerosis Board of American Heart Association [2].

Exteriorly unchanged segments of aorta had a smooth luminal surface. On vertical section in intima, it was possible to detect 2 layers: adjacent to aorta opening proteoglycan layer and an adjoining media myoelastic layer.

Segments of intima with primary lesions (lesion type I) were macroscopically segments with smooth yellowish surface, sometimes with small yellow spots. Microscopical changes were minimal. Small accumulations of extracellular lipidic drops in connective tissue matrix were observed. Along with tissue-fixed cells, in segments of preliminary lesions, there were more mononuclear cells compared to normal intima. There were no changes in tissue structure detected.

Fatty streaks (lesion type II) macroscopically were stripes and spots of yellow colour, slightly standing out over the vessel surface. Fatty streaks often merge together forming larger structures (clusters). In tissue sections, lipids were detected mainly intracellularly. Intracellular lipids were also detected in connective tissue matrix. Sometimes there can be observed an excessive overgrowth of extracellular matrix in fatty streaks.

During macroscopic investigation, lipofibrous plaques (lesion type Va) looked like yellowish or pearl round or ellipsoidal formations standing far out over luminal surface. Microscopically in these lesions, all the changes were detected, which are characteristic for fatty streaks: accumulation of intracellular lipids and overgrowth of extracellular matrix. Furthermore, there were a massive necrotic nucleus and a connective tissue cap found in lipofibrous plaques. In lipofibrous plaques, there were such segments, which are morphologically similar to segments of lipidic lesions, arms of lipofibrous plaques.

Fibrous plaques (lesion type Vc) are macroscopically highly elevated round and oval formations of pearl colour, microscopically consisting mainly of coarse connective tissue matrix with cells “mured” in the matrix.

Aortic intima was morphologically mapped according to the presence of atherosclerotic lesions of varying severity or healthy vascular tissue.

**Figure 1:**
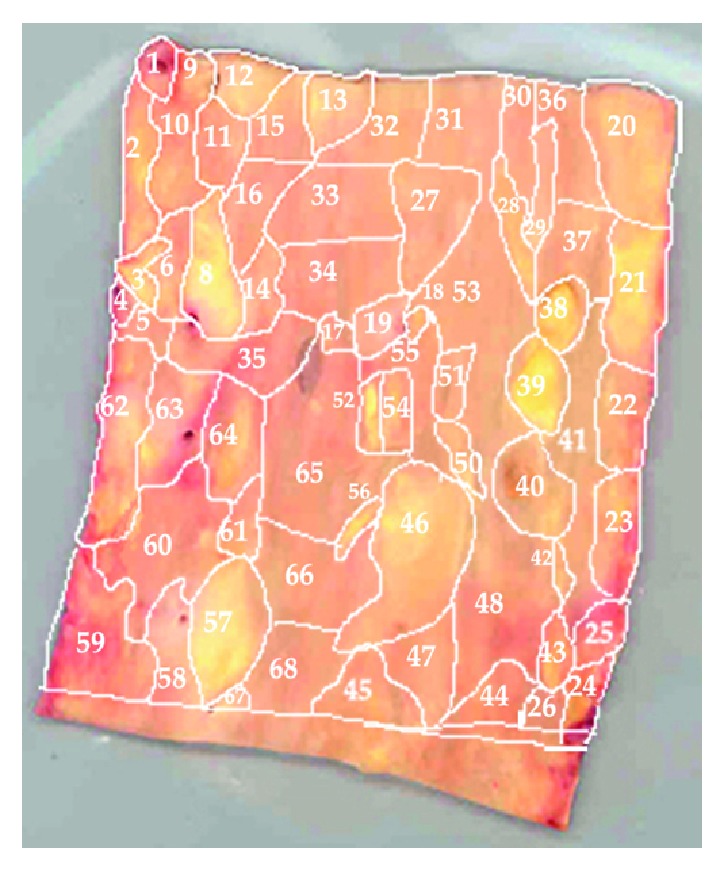
Morphological map of aorta number 1.

**Figure 2:**
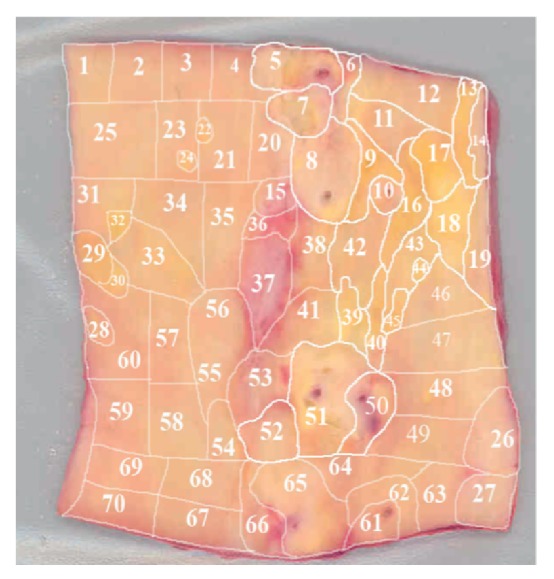
Morphological map of aorta number 2.

**Figure 3:**
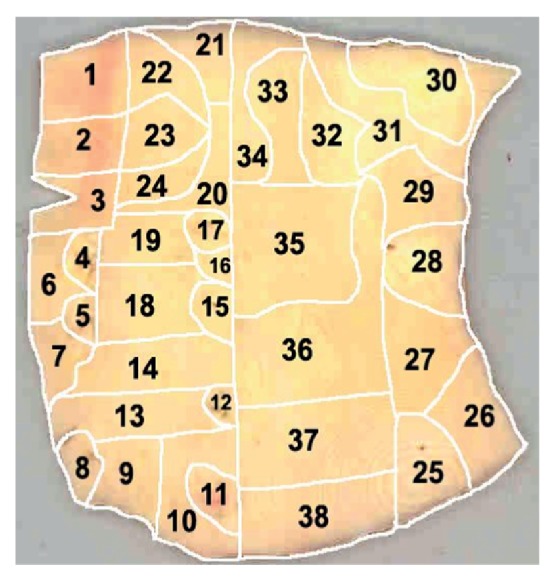
Morphological map of aorta number 3.

**Figure 4:**
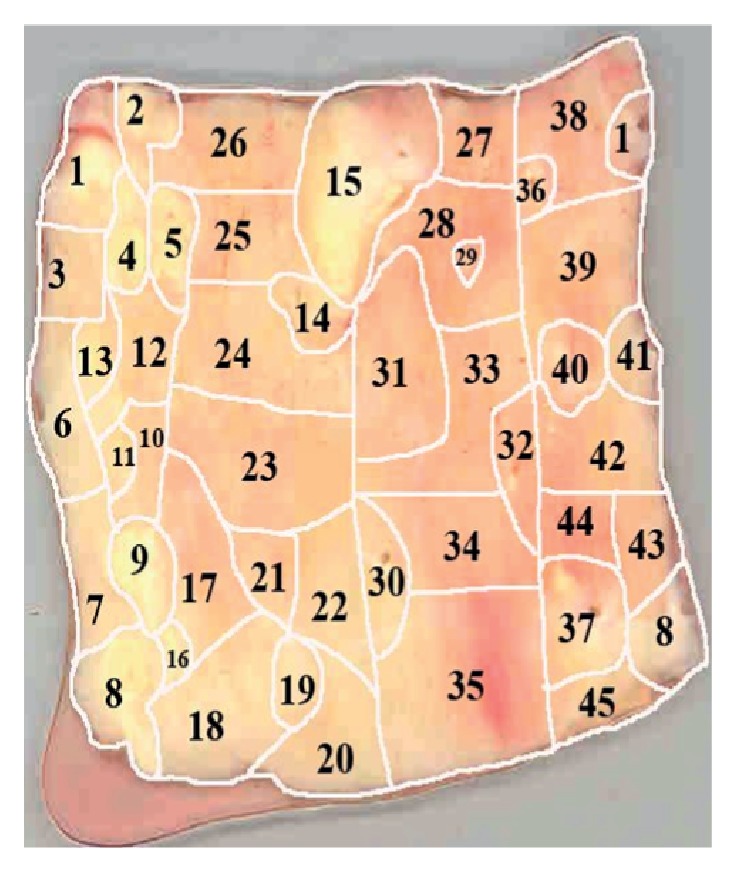
Morphological map of aorta number 4.

**Figure 5:**
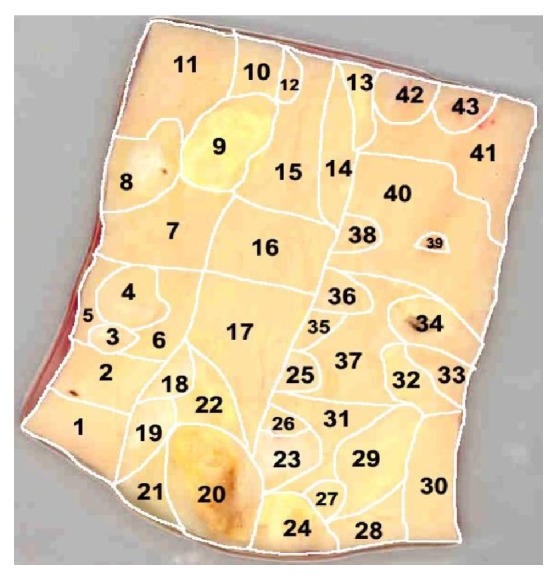
Morphological map of aorta number 5.

*Morphological Mapping of Aortas*. In this study, 5 aortic samples were used. Each sample was a segment of a vascular wall with a size approximately 7 × 9 cm^2^, divided according to morphological characteristics into regions with atherosclerotic lesions of varying severity (1—normal tissue, 2—fatty infiltration, 3—fatty streak, 4—lipofibrous plaque, 5—fibrous plaque). In each aortic sample, 38 to 70 of such regions were identified (Figures 1, 2, 3, 4, and 5). In total, 265 segments of aortic intima were analyzed (Table 1).

Morphological maps for each aortic sample were made and in each morphological map the relative positions of the identified regions were marked. Morphological maps of investigated aortas are presented on Figures 1–5.

Furthermore, an opportunity of making a broader classification of regions was taken: normal tissue, early lesions (combination 2+3), and late lesions (combination 4+5).

### 2.2. Methods

#### 2.2.1. DNA Isolation

Isolation of total DNA from aortic tissue samples (10 *μ*g) was carried out using the phenol-chloroform extraction with the proteinase K lysis. The concentration of the DNA solution was measured by nanospectrophotometer IMPLEN NanoPhotometer at a wavelength of 260 nm.

#### 2.2.2. PCR

For PCR, we used DNA with concentration 0.1 *μ*g/mL and primers with concentration 10 pmol/*μ*L. Sequences of primers for PCR are presented in Supplemental Table 1 (see Supplemental Table 1 in Supplementary Material available online at http://dx.doi.org/10.1155/2014/825468).

A reaction mixture and PCR conditions were as follows:

 MQ (H_2_O)-4.6 *μ*L;
 dNTPs mixture 10X: 2 mM dATP, 2 mM dTTP, 2 mM dGTP, and 2 mM dCTP-4 *μ*L;
 10X buffer (16.6 *μ*M (NH_4_)_2_SO_4_, 67 mM Tris-HCl (pH 8.8)-4 *μ*L);
 MgCl_2_: 25 mМ
  (i) 4 *μ*L (the required concentration is 2.5 mM);
  (ii) 2.4 *μ*L (the required concentration is 1.5 mM);
 Taq- polymerase (“Syntol,” Russia)-1.33 *μ*L
 DNA template-4 *μ*L;
 primer F (+)-2.7 *μ*L;
 primer R (−)-2.7 *μ*L.

The reaction was carried out in 40 *μ*L volume.

Conditions for PCR are presented in Supplemental Table 2.

#### 2.2.3. Electrophoresis of DNA Samples and PCR-Fragments

An electrophoresis of extracted DNA samples and PCR-fragments was performed in horizontal apparatus (“Helicon,” Russia) in agarose gel with the use of 0.5Х ТBЕ buffer. The concentration of agarose (≪Fluka≫) was 0.8% (for DNA samples) and 1.5–2.0% (for PCR-fragments).

One of the primers was biotinylated with the aim of further pyrosequencing of PCR-fragment. The investigation was performed with the use of an amplifier ≪PTC DNA Engine 200≫.

The examples of gel electrophoresis of PCR-fragments of investigated mitochondrial mutations are presented in Supplemental Figures 1–4.

#### 2.2.4. Pyrosequencing

After carrying out PCR, the PCR-fragments were pyrosequenced to detect point substitutions, microinsertions, or microdeletions of human mitochondrial genome. The investigation was performed with an automatic pyrosequencing system PSQ HS96MA. During the experiment, a scheme of sample conditioning, described in a manual attached to the pyrosequenator, was realized (sepharose particles were used). Sequences of primers for sequencing are listed in Supplemental Table 3.

A visualization of results was performed using a software attached to the pyrosequencing system. The statistical evaluation of the results was performed by using SPSS version 21.0.

## 3. Results

For assessment of 11 mitochondrial mutations, identified as potential atherosclerosis markers, an investigation on autopsy material with morphological and mutational mapping was made.

### 3.1. Analysis of 11 Mitochondrial Genome Mutations

During the analysis of all the segments of normal and atherosclerotic intima of 5 aortas by means of bootstrap analysis, it was found that total atherosclerotic lesion of mapped aortas is positively associated with mitochondrial genome mutations C3256T, T3336C, C5178A, G12315A, G14459A, and G15059A (significantly) and mutation 652delG is at *P* ≤ 0.1 level of significance. Meanwhile, mutation G13513A had a significantly negative association with total atherosclerotic lesion of mapped aortas (Table 2).

According to Wilcoxon Matched-Pairs Signed-Ranks test (on averaged data, it is for all the aortas simultaneously), it was found (Table 3) that mitochondrial genome mutations, characteristic for total atherosclerotic lesion, are associated at the same level of significance with primary total atherosclerotic lesion (lipidic spots and fatty streaks) and a total of all the segments of lipofibrous plaques. Mutation G13513A correlates with these types of total lesion significantly negatively. Moreover, mutation A1555G negatively correlates with primary total atherosclerotic lesion and a total of segments of lipofibrous plaques at *P* ≤ 0.05 level of significance. During analyzing a total of segments of lipofibrous plaques, a significantly negative correlation of mutation G14846A with this type of lesion was found.

At the same time, there was found a positive correlation between a total of segments of lipofibrous and fibrous plaques and mitochondrial genome mutation 652delG at *P* ≤ 0.1 level of significance. However, this mutation is absent in primary total atherosclerotic lesion.

Moreover, a total of fibrous plaque segments correlated at *P* ≤ 0.05 level of significance with mutations C3256T and C5178A (positively) and G12315A (negatively).

### 3.2. Cumulative Mutational Burden of 11 Mutations in Morphologically Mapped Aortas

To detect the presence of mutational burden interrelation with the degree of atherosclerotic lesion, a linear regression analysis was carried out. Because of a high individual variability of the characteristic, these values were normalized as quartiles. In every aorta for each mutation, an investigation of heteroplasmy index distribution was carried out and interquartile boundaries were detected. Individual scalar indexes of heteroplasmy were transformed into single values 1, 2, 3, or 4, characterizing a belonging of an index to a certain quartile within the limits of a given autopsy sample. The results are presented in Tables 4 and 5.

Therefore, during the assessment of cumulative burden for 11 mutations, the model of linear regression reached *P* < 0.001 level of significance. Taking into consideration a sufficient quantity of degrees of freedom (11), it can therefore be said that the degree of atherosclerotic lesion is associated with cumulative burden for these mutations with 99.9% probability of error-free prognosis.

**Figure 6:**
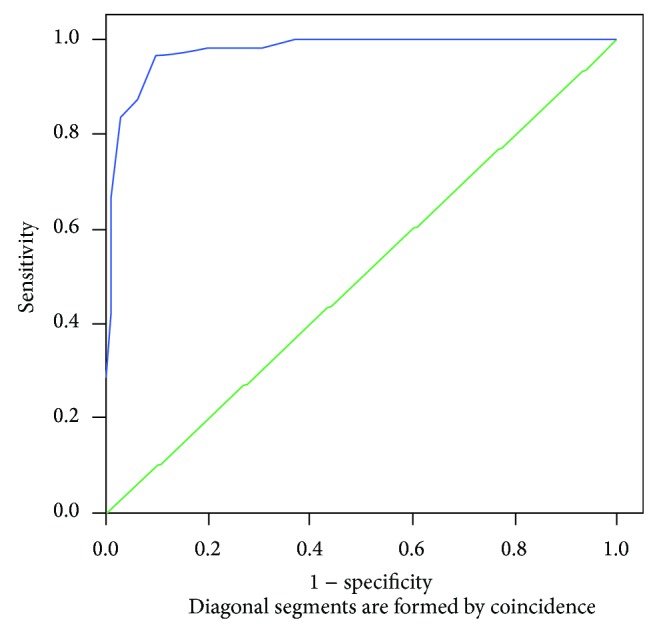
ROC-curve for assessment of sensitivity and specificity of an index “mutational burden” concerning atherosclerosis. Positive real state is an atherosclerotic plaque. The area under the curve is 0.975 (*P* < 0.001).

For each mutation associated with atherosclerosis, sensitivity and specificity indexes were studied. The analysis was carried out by a method of ROC-curve constructing with a further evaluation of the area under the curve. This enabled us to describe explanatory characteristics of genotypic markers (Figure 6).

For this analysis, rank values (quartile numbers) of heteroplasmy were summed according to a sign of beta coefficient, obtained during regression analysis (if the value of a coefficient was positive, addition was done; if the value was negative, subtraction was done). The obtained parameter was named “mutational burden.”

During the model using, the sensitivity index was 88.2 (*P* ≤ 0.05, because a 95% confidence interval lies in the range from 74.6 to 95.3). Specificity index was 77.1 (*P* ≤ 0.05, because a 95% confidence interval lies in the range from 70.8 to 87.3).

Therefore, cumulative mutational burden for 11 investigated mitochondrial genome mutations is associated with 88.2% of cases of atherosclerotic lesions in morphologically mapped aortas.

## 4. Discussion

Blood cells play a great role in the origin and development of atherosclerotic lesions in arterial intima. In atherogenesis, they migrate through endothelium and intima-medial layer of vessels. Meanwhile, lymphocytes play a signaling role in immune and inflammatory response formation and monocytes form macrophage cells, aimed at removing the abundance of cholesterol, accumulated in the atherosclerotic lesion focus. A probable role of mitochondrial genome mutations in the origin and development of atherosclerosis may be the fact that these mutations result in protein chain defects in respiratory chain enzymes of mitochondria or transfer RNA. Meanwhile, a metabolism level of defective mitochondria lowers and as a result monocytes containing them acquire a liability to lipoidosis. Therefore, a result of pathophysiologic processes, started by mitochondrial genome mutations, is a transformation of mutant monocytes into foam cells.

To test this hypothesis, a decision to carry out a comparative analysis of heteroplasmy level in normal and affected by atherosclerosis human arterial intima was made.

To detect a heteroplasmy level in the samples investigated, a new original method of quantitative assessment of the mutant allele of mitochondrial genome [22, 23], based on pyrosequencing technology, was developed by the authors [24]. This method is suitable for the study of any biological samples. With its help, it is possible to detect the heteroplasmy level of both hereditary and somatic mitochondrial genome mutations which appear during the life of an individual or in pathological processes. Furthermore, it was possible to detect the percentage of nuclear genome somatic mutations, occurring, for example, in the course of occurrence and development of cancer processes.

In the present study, for the first time we obtained data, demonstrating that different segments of the aortic intima, both normal and having atherosclerotic lesions of varying severity, may differ in heteroplasmy level of mutant allele of mitochondrial genome. According to the literature, the investigated mutations are associated with different pathologies (Table 6).

Most types of aortic atherosclerotic lesions turned out to be associated with mutations C3256T, T3336C, C5178A, G12315A, G14459A, and G15059A, which may indicate that the key point in the start of pathophysiological mechanisms, which result in atherosclerotic lesions formation in human aortas, is defects in transport RNA-Leu (codons recognized UUR and CUN) and also 1, 2, and 6, NADH-dehydrogenase subunit, and cytochrome B.

Conspicuous is the fact that the mutation spectrum in different types of atherosclerotic lesions of the aortic intima may slightly differ. For example, mutation A1555G showed an antiatherogenic effect in early atherosclerotic lesions (lipidic spots and fatty streaks) and lipofibrous plaques. At the same time, antiatherogenic mutation G14846A turned out to be typical only for lipofibrous plaques.

In fibrous plaques, mutation spectrum significantly differed from other types of atherosclerotic lesions. Proatherogenic mutations were C5178A and C3256T; antiatherogenic mutation was G12315A. Apparently, the defective parts in G12315A transport RNA-Leu (codon recognized CUN) play an important role in pathophysiological process of the origin and development of atherosclerotic lesions before the fibrous plaque stage begins, when the percentage of heteroplasmy for this mutation decreases to such an extent that it is higher in normal intima than in the type of atherosclerotic lesion. The heteroplasmy level of some other mitochondrial genome mutations in fibrous plaques apparently decreases and does not vary from this parameter in normal aortic intima.

## 5. Conclusion

In the present study, a focality of atherosclerotic lesions in human aortic intima was confirmed. The basis for this confirmation was differences in the heteroplasmy level of 11 mitochondrial mutations for different segments of both normal and atherosclerotic morphologically mapped human aortic intima. It was found that a certain spectrum of pro- and antiatherogenic mitochondrial genome mutations is characteristic for different types of atherosclerotic lesions of aortic intima. In total atherosclerotic lesion, lipofibrous plaques, and primary atherosclerotic lesions of the aortic intima, six proatherogenic mutations (C3256T, T3336C, C5178A, G12315A, G14459A, and G15059A) and one antiatherogenic mutation (G13513A) were identified. Differences in the spectrum of mutations between primary atherosclerotic lesions, lipofibrous plaques, and total atherosclerotic lesions lie in the fact that, for the first two types, antiatherogenic mutation A1555G is representative. In lipofibrous plaques, mutation G14846A is also antiatherogenic. The mutation spectrum in fibrous plaques is different from other types of atherosclerotic lesions. Proatherogenic mutations in fibrous plaques are C5178A and C3256T. Mutation G12315A is antiatherogenic.

The findings of this study may be useful for practitioners and medical geneticists for early detection and family analysis of atherosclerosis.

## Supplementary Material

Supplemental material provides the data about gel/electrophoresis of PCR-fragments, containing mutation sites (Supplementary FIGURES 1-4), primers for PCR (Supplementary TABLE 1), the conditions for the PCR of the mitochondrial genome fragments (Supplementary TABLE 2), and primers for pyrosequencing (Supplementary TABLE 3).

## Figures and Tables

**Table 1:** Atherosclerotic lesion degree of morphologically mapped aortas.

Number of aorta	Lesion degree	Quantity of segments
1	Normal tissue	9
	Fatty infiltration	17
	Fatty streak	6
	Lipofibrous plaque	5
	Fibrous plaque	1

2	Normal tissue	13
	Fatty infiltration	10
	Fatty streak	7
	Lipofibrous plaque	12
	Fibrous plaque	3

3	Normal tissue	12
	Fatty infiltration	6
	Fatty streak	6
	Lipofibrous plaque	12
	Fibrous plaque	7

4	Normal tissue	15
	Fatty infiltration	14
	Fatty streak	18
	Lipofibrous plaque	12
	Fibrous plaque	9

5	Normal tissue	25
	Fatty infiltration	17
	Fatty streak	13
	Lipofibrous plaque	10
	Fibrous plaque	5

**Table 2:** Bootstrap analysis of a correlation coefficient between a heteroplasmy level and the presence of a total atherosclerotic lesion of mapped aortas.

Mutation	Correlation coefficient value	Asymptotical significance (2-tailed)
652delG	0.311^*^	0.088^*^
652insG	−0.301	0.121
A1555G	−0.307	0.113
C3256T	0.353^**^	0.050^**^
T3336C	0.439^**^	0.023^**^
C5178A	0.357^**^	0.047^**^
G12315A	0.403^**^	0.045^**^
G13513A	−0.456^**^	0.035^**^
G14459A	0.453^**^	0.036^**^
G14846A	−0.297	0.129
G15059A	0.451^**^	0.037^**^

^*^Correlation of mutations with atherosclerotic lesions at *P* ≤ 0.1 level of significance; ^**^significant correlation of mutations with atherosclerotic lesions (*P* ≤ 0.05).

**Table 3:** Major mitochondrial genome mutations in different types of total atherosclerotic lesion of morphologically mapped aortas.

Mutations	Primary total atherosclerotic lesion		Lipofibrous plaques		Fibrous plaques	
	Correlation coefficient	Asymptomatic significance	Correlation coefficient	Asymptomatic significance	Correlation coefficient	Asymptomatic significance
652delG	0.093	0.146	0.308^*^	0.091^*^	0.323^*^	0.071^*^
652insG	−0.075	0.186	−0.095	0.125	−0.058	0.232
A1555G	−0.359^**^	0.048^**^	−0.401^**^	0.039^**^	−0.084	0.195
C3256T	0.368^**^	0.045^**^	0.407^**^	0.045^**^	0.352^**^	0.050^**^
T3336C	0.426^**^	0.034^**^	0.437^**^	0.025^**^	0.103	0.119
C5178A	0.365^**^	0.046^**^	0.439^**^	0.023^**^	0.356^**^	0.048^**^
G12315A	0.353^**^	0.050^**^	0.409^**^	0.041^**^	−0.367^**^	0.046^**^
G13513A	−0.423^**^	0.035^**^	−0.437^**^	0.021^**^	0.095	0.143
G14459A	0.403^**^	0.042^**^	0.463^**^	0.026^**^	0.073	0.191
G14846A	−0.107	0.117	−0.351^**^	0.050^**^	0.052	0.214
G15059A	0.405^**^	0.043^**^	0.471^**^	0.015^**^	0.062	0.203

^*^Correlation of mutations with atherosclerotic lesions at *P* ≤ 0.1 level of significance; ^**^significant correlation of mutations with atherosclerotic lesions (*P* ≤ 0.05).

**Table 4:** Summary of a linear regression model of mutational burden with a degree of atherosclerosis in aortas.

Model	*R*	*R* ^2^	Corrected *R* ^2^	Standard error of estimation
1	0.945	0.894	0.886	0.278

Note that predictors of the model were a constant, quartiles of G14846A, quartiles of 625delG, quartiles of T3336C, quartiles of C5178A, quartiles of A1555G, quartiles of G14459A, quartiles of G15059A, quartiles of 625insG, quartiles of G12315A, quartiles of G13513A, and quartiles of C3256T.

**Table 5:** Dispersion analysis of linear regression model of mutational burden with a degree of atherosclerosis.

Model		Sum of squares	Degrees of freedom	Mean square	*F*	Significance
1	Regression	93.8	11	8.53	110.0	<0.001
	Residual	11.2	144	0.08		
	Total	**104.9**	**155**			

Note that predictors of the model were a constant, quartiles of G14846A, quartiles of 625delG, quartiles of T3336C, quartiles of C5178A, quartiles of A1555G, quartiles of G14459A, quartiles of G15059A, quartiles of 625insG, quartiles of G12315A, quartiles of G13513A, and quartiles of C3256T.

**Table 6:** Data on the pathologies, caused by the investigated mutations.

Gene	Mutation	Pathology
Gene 12S rRNA	652insG	Gastric carcinoma [25]
	A1555G	Dullness of hearing, induced by aminoglycosides and idiopathic hearing loss, sensibility to aminoglycoside antibiotics; deafness [26–28]

Gene tRNA-Leu (codon recognized UUR)	C3256T	MELAS, encephalopathy, lactic acidosis, myopathy, cardiomyopathy, stroke-like lesion in the right parietooccipital brain region, and oxidative defect of muscular metabolism [8]

Gene of subunit 1 NADH dehydrogenase	T3336C, a silencing mutation	Type 2 diabetes mellitus [29]

Gene of subunit 2 NADH dehydrogenase	C5178A causes a substitution of leucine for methionine	Acute myocardial infarction [30]

Gene tRNA-Leu (codon recognized CUN)	G12315A	Encephalopathy [31]

Gene of subunit 5 NADH dehydrogenase	G13513A	Li syndrome (hereditary encephalomyopathy), Wolff-Parkinson-White syndrome (preexcitation syndrome), and cardiomyopathy [7, 32]

Gene of subunit 6 NADH dehydrogenase	G14459A (a substitution of alanine for valine in the seventy-second amino acid position, which is located in the most conservative region of protein ND6)	Hereditary Leber's optic atrophy. Associated with dysfunction of basal ganglia, muscular spasticity, and encephalopathy [33, 34]

Gene of cytochrome B	G14846A (a substitution of glycine for serine in position 34 (G34S) that weakens enzymatic function of cytochrome B)	Mitochondrial myopathies [35]
	G15059A (a nonsense mutation, as the result of which amino acid glycine in position 190 is substituted for a terminating codon, causing a stopping in translation, protein size reduction, and a loss of 244 amino acids from C-terminus of a protein). The mutation weakens enzyme function of cytochrome B.	Mitochondrial myopathies [35]
