# Supplementary material for: Mosaicism of Mitochondrial Genetic Variation in Atherosclerotic Lesions of the Human Aorta
Source: Biomed Res Int. 2015 Mar 5;2015:825468. doi: 10.1155/2015/825468 (PMC4365331; doi:10.1155/2015/825468)
Supplement: Supplementary file 1 — Supplemental material provides the data about gel/electrophoresis of PCR-fragments, containing mutation sites (Supplementary FIGURES 1-4), primers for PCR (Supplementary TABLE 1), the conditions for the PCR of the mitochondrial genome fragments (Supplementary TABLE 2), and primers for pyrosequencing (Supplementary TABLE 3). [file 825468.f1.pdf]

Supplementary material

Supplemental material provides the data about gel/electrophoresis of PCR-fragments, containing mutation sites (**Supplementary FIGURES 1-4**), primers for PCR (**Supplementary TABLE 1**), the conditions for the PCR of the mitochondrial genome fragments (**Supplementary TABLE 2**), and primers for pyrosequencing (**Supplementary TABLE 3**).

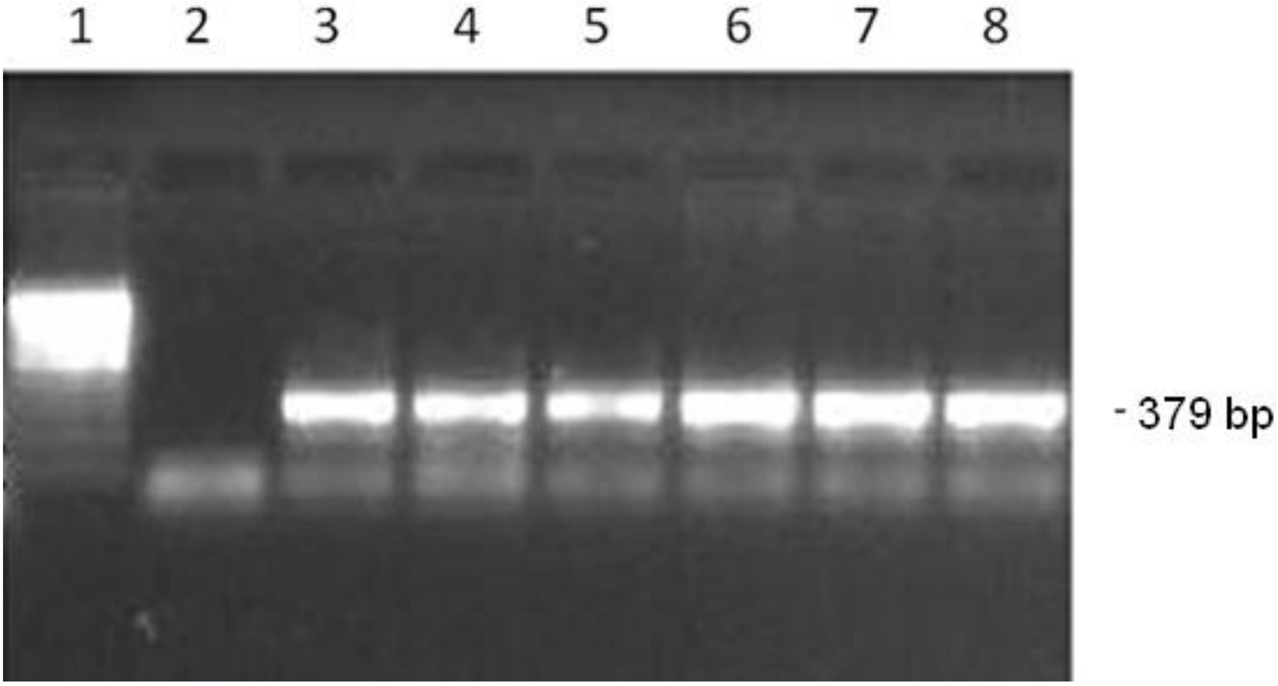

Supplementary FIGURE 1. Gel/electrophoresis of PCR-fragments, containing mutation site A1555G:

1. DNA marker 100 bp (10 fragments from 100 to 1000 bp);

2. Negative Control;

3-8. PCR-fragments, containing mutation site A1555G.

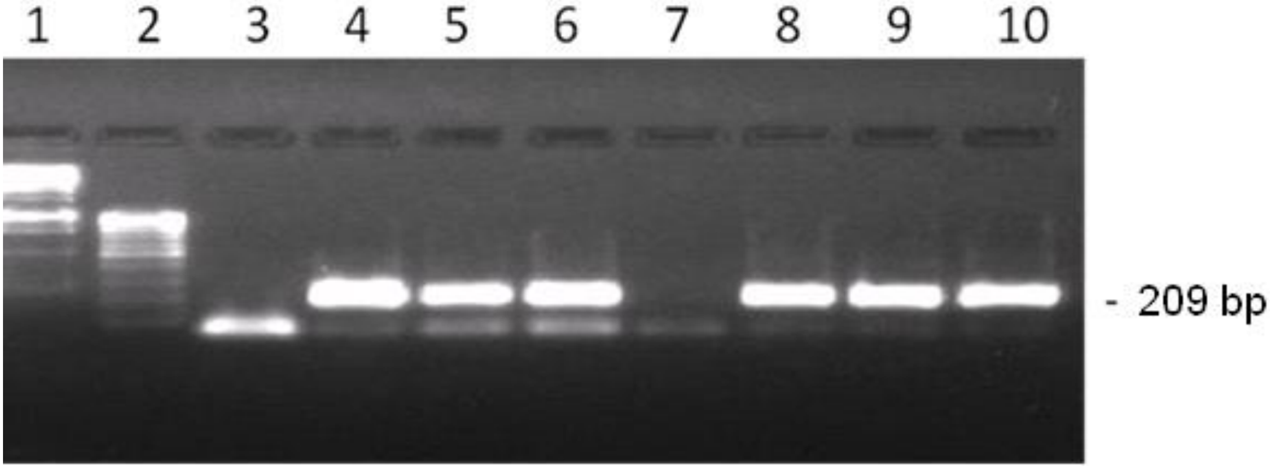

Supplementary FIGURE 2. Gel/ electrophoresis of PCR-fragments, containing mutation site G14459A:

1. DNA marker 1 Kb (13 fragments from 0.25 to 10 kb);

2. DNA marker 100 bp (10 fragments from 100 to 1000 bp);

3 и 7. Negative Control;

4-6 и 8-10. PCR-fragments, containing mutation site G14459A.

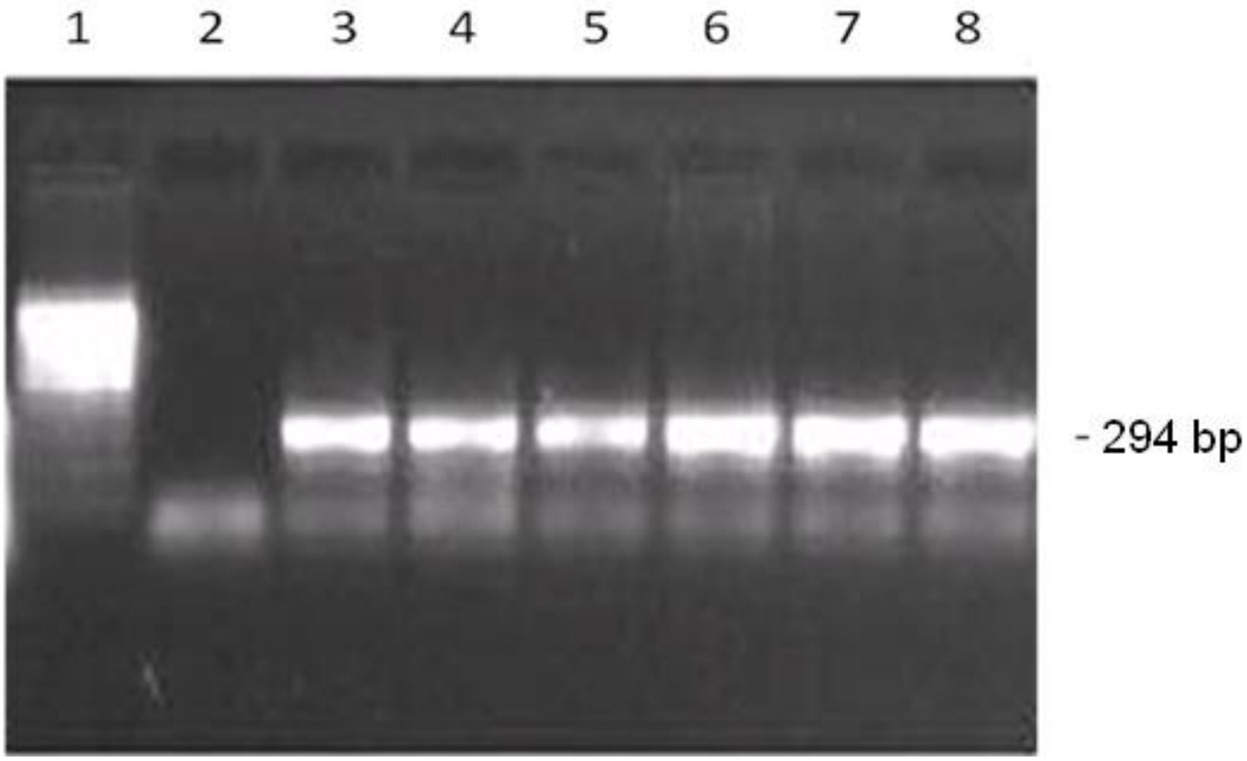

Supplementary FIGURE 3. Gel/electrophoresis of PCR-fragments, containing mutation sites C3256T and T3336C:

1. DNA marker 100 bp (10 fragments from 100 to 1000 bp);

2. Negative Control;

3-8. PCR-fragments, containing mutation sites C3256T and T3336C.

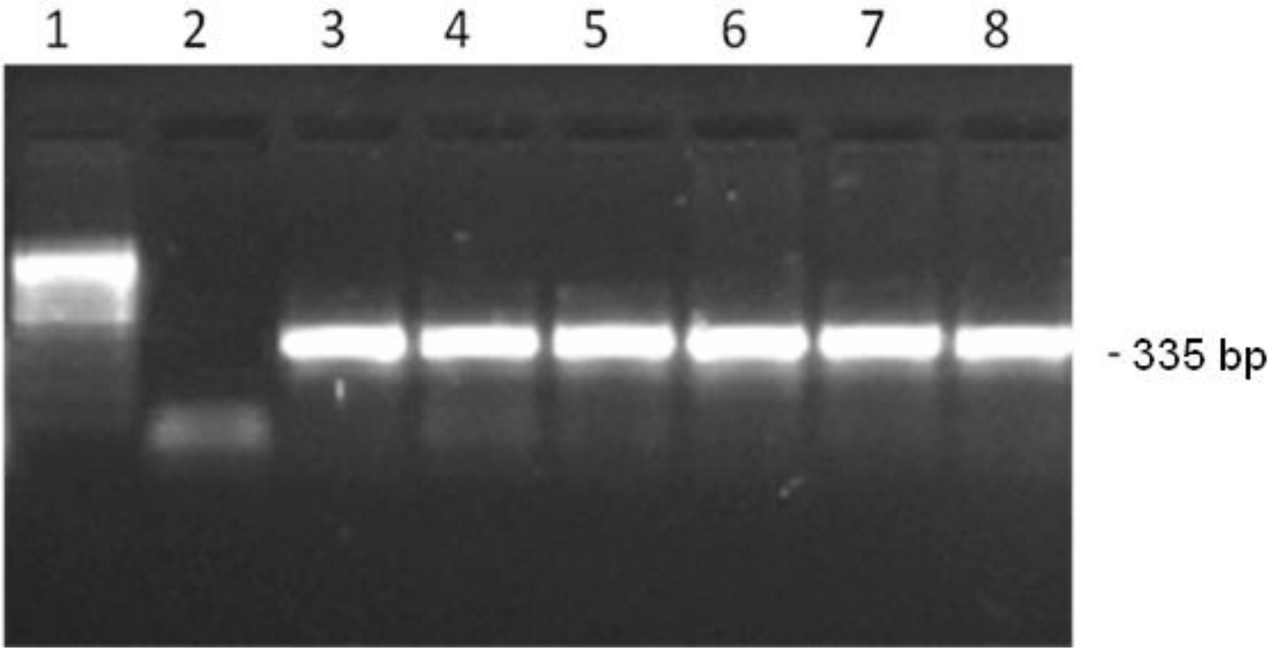

Supplementary FIGURE 4. Gel/electrophoresis of PCR-fragments, containing mutation site G13513A:

1. DNA marker 100 bp (10 fragments from 100 to 1000 bp);

2. Negative Control;

3-8. PCR-fragments, containing mutation sites G13513A.

Supplementary TABLE 1. Primers for PCR

Mutation	Forward primer for PCR	Reverse primer for PCR
652delG	TAGACGGGCTCACATCAC (621 - 638)	bio-GGGGTATCTAATCCCAGTTTGGGT (1087 - 1064)
652insG		
A1555G	TAGGTCAAGGTGTAGCCCATGAGG TGGCAA(1326 - 1355)	bio-GTAAGGTGGAGTGGGTTTGGG (1704 - 1684)
C3256T	bio-AGGACAAGAGAAATAAGGCC (3129 - 3149)	ACGTTGGGGCCTTTGCGTAG (3422 - 3403)
T3336C		
C5178A	bio-GCAGTTGAGGTGGATTAAAC (4963 - 4982)	GGAGTAGATTAGGCGTAGGTAG (5366 - 5345)
G12315A	bio-CTCATGCCCCCATGTCTAA (12230 – 12249)	TTACTTTTATTTGGAGTTGCAC (12337 -12317)
G13513A	CCTCACAGGTTTCTACTCCAAA (13491 – 13512)	bio-AAGTCCTAGGAAAGTGACAGCGA GG(13825 - 13806)
G14459A	CAGCTTCCTACACTATTAAAGT (14303 – 14334)	bio-GTTTTTTTAATTTATTTAGGGGG (14511 – 14489)
G14846A, G15059A	bio-CATTATTCTCGCACGGACT (14671 – 14689)	GCTATAGTTGCAAGCAGGAG (15120 – 15100)

Supplementary TABLE 2. Conditions for the PCR of the mitochondrial genome fragments.

Mutations	Size of PCR-fragment	Concentration of $MgCl_2$ in PCR-buffer	Denaturation	Annealing	Extension
652delG, 652insG	467 bp	2.5 мМ	94^0	60^0	72^0
C5178A	383 bp				
C3256T, T3336C	294 bp	2.5 мМ	94^0	55^0	72^0
G13513A	335 bp	1.5 мМ			
G14846A, G15059A	450 bp				
A1555G	379 bp	2.5 мМ	94^0	50^0	72^0
G12315A	108 bp	2.5 мМ			
G14459A	209 bp	1.5 мМ			

Supplementary TABLE 3. Primers for pyrosequencing*.

**Mutation**	**Primer for pyrosequencing**
652insG	CCCATAAACAAATA
A1555G	ACGCATTTATATAGAGGA
C3256T	AAGAAGAGGAATTGA
652delG	CCCATAAACAAATA
T3336C	TGCGATTAGAATGGGTAC
C5178A	ATTAAGGGTGTTAGTCATGT
G12315A	TTTGGAGTTGCAC
G13513A	AGGTTTCTACTCCAA
G14459A	GATACTCCTCAATAGCCA
G14846A	GCGCCAAGGAGTGA
G15059A	TTTCTGAGTAGAGAAATGAT

*A selection of primers was made using of Primer3 software.
